# Miniature 3D-Printed Centrifugal Pump with Non-Contact Electromagnetic Actuation

**DOI:** 10.3390/mi10100631

**Published:** 2019-09-21

**Authors:** Luca Joswig, Michael J. Vellekoop, Frieder Lucklum

**Affiliations:** Institute for Microsensors, Actuators and Systems (IMSAS), Microsystems Center Bremen (MCB), University of Bremen, D-28359 Bremen, Germany; ljoswig@uni-bremen.de (L.J.); mvellekoop@imsas.uni-bremen.de (M.J.V.)

**Keywords:** 3D-printed pump, centrifugal pump, electromagnetic actuation, integrated magnets

## Abstract

We present a miniature 3D-printed dynamic pump using the centrifugal operating principle. Dynamic pumps typically yield higher flow rates than displacement pumps at reasonable output pressure. Realizing smaller devices suitable for millifluidic and microfluidic applications brings challenges in terms of design, fabrication and actuation. By using microstereolithography printing we have reduced the overall size to an effective pumping volume of 2.58 mL. The free-moving rotor consists of an impeller and permanent magnets embedded during the printing process, which allow for non-contact electromagnetic actuation. The pump is driven by periodically switching the current through stator coils, controlled by a custom built circuit using a Hall effect sensor. It achieves a maximum flow rate of 124 mL/min and a hydrostatic pressure of up to 2400 Pa.

## 1. Introduction

Pumps are a major component of many experiments using microfluidic devices. Despite years of research on different pumping mechanisms suitable for the microscale [1,2], large commercial pumps are still utilized for most laboratory setups, or the microdevices are filled manually via a syringe or passively by capillary forces from a droplet or reservoir. Micropumps are also usually limited by a relatively low flow rate due to their size and operation principles. On the other hand, using macroscopic pumping mechanisms on smaller scales is often challenging due to difficulties in design and fabrication. Working towards miniature pumps that offer high flow rates is a topic of ongoing research and development.

Additive manufacturing, commonly known as 3D-printing, can be used to address many challenges when working with microfluidic devices. The increased spatial freedom nowadays allows revolutionary new designs and approaches to microfluidcs [3,4,5] with functional feature sizes covering the centimeter, millimeter and sub-millimeter, down to the micron and even sub-micron scale. Our group recently highlighted the possibilities and solutions for microfluidic chip-to-world connections [6], addressing the challenges of suitable packaging for microfluidic chips requiring, for example, electrical, fluidic and optical access. Additionally, we realized miniature printed fluidic components with integrated free-moving permanent magnets as, for example, active check-valves, fluid dispensers and mixers. In recent years, a number of 3D-printed fluidic pumps using different mechanisms have been proposed. For example, Au et al., demonstrated pneumatic membrane valves that could be combined into a peristaltic pump 3D-printed from a photopolymer resin [7]. Gong et al., presented a comparable but smaller printed membrane pump also using external pneumatic actuation [8]. Habhab et al., fabricated a miniature rotary pump based on the Tesla turbine principle and externally driven by a magnetic stirrer [9]. Another useful component of a fluidic system is a flow sensor to accurately determine the flow rate, such as a 3D-printed rotary impeller using a ferromagnetic material [10].

In this work, we present a miniature 3D-printed centrifugal pump that achieves high flow rates for millifluidic and microfluidic applications. The internal rotor utilizes permanent magnets integrated during the printing process and thus allows non-contact electromagnetic coupling to stator coils outside the pump, resembling the driving principle of a brushless direct current (DC) motor [11].

## 2. Materials and Methods

### 2.1. Centrifugal Pumping Principle

In contrast to positive displacement pumps, centrifugal (or rotodynamic) pumps impart kinetic energy from a rotor or impeller to the liquid, therefore producing flow by creating a pressure gradient between inlet and outlet [12]. Centrifugal pumps are commonly used to pump low viscosity fluids in high flow rate, low pressure applications. They only have a single moving component and are therefore robust and versatile. The overall pump dimensions relative to its output are also very compact in comparison to other pump designs.

**Figure 1:**
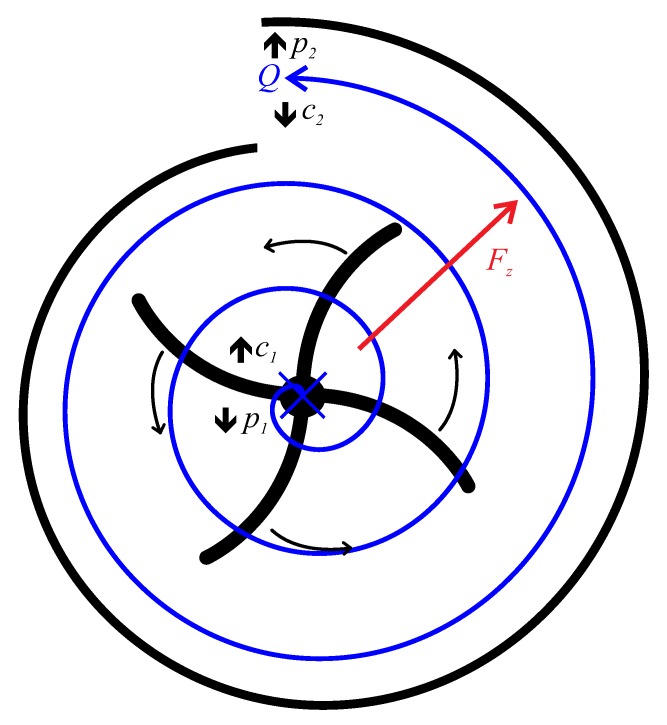
Centrifugal pumping principle with fluid flow *Q* (blue arrow) coming from top, rotated by impeller (black) and pushed outwards due to centrifugal force $F_{z}$ (red) into spiral shape of pump chamber, creating a pressure gradient from inlet to outlet $p_{2}>p_{1}$.

Figure 1 illustrates the working principle of a centrifugal pump based on our design. Liquid flows from the axial inlet atop the impeller to a tangentially oriented outlet. Due to the rotating impeller the liquid is accelerated into a circular motion and pressed radially outwards because of centrifugal forces $F_{z}$. The pump chamber has an equivalent spiral shape (volute) for the active compartment. Following the continuity equation for incompressible liquids and the Bernoulli equation, this spiral shape leads to a pressure gradient from inlet to outlet, $p_{2}>p_{1}$, due to the increase in cross-sectional area and a corresponding decrease in flow velocity, $c_{2}<c_{1}$. Consequently, an overall fluid flow *Q* is produced between inlet and outlet.

### 2.2. Pump Design

**Figure 2:**
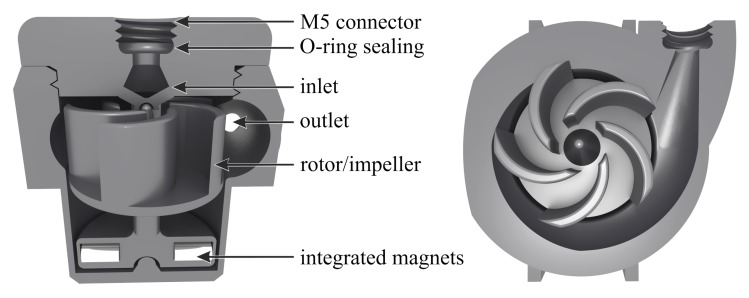
Perspective cut view from side (**left**) and top (**right**) of 3D-printed centrifugal pump design consisting of three separate parts: rotor with embedded permanent magnets (2 mm × 4 mm) and impeller blades, pump housing with outlet connector and inlet lid. The overall width, depth and height of the pump is 28 mm × 30 mm × 24 mm, respectively.

We designed our centrifugal pump to consist of three separate parts, namely free-moving rotor, pump housing and inlet lid. The rotor sits inside the housing centered by an upper and lower bearing. It encompasses a lower cylindrical element, into which we embed four permanent magnets for the electromagnetic actuation by an outside stator and an impeller with five blades sitting on a thin platform at the top. The impeller is a semi open design as a compromise between simplicity, robustness and performance. The rotor diameter is 15 mm and its height is 18 mm. The rotor axle ends in a rounded tip at the top and half-sphere cup at the bottom, which allows for relatively low friction contact with corresponding half-sphere bearings. The different margins between rotor, bearing and pump housing are as low as 200 µm, with minimal wall thicknesses as low as 300 µm. An inlet lid is screwed to the top of the pump housing after insertion of the rotor, with a custom thread design allowing airtight sealing. The inlet and outlet support the insertion of a 4 mm O-ring (2 mm inner diameter) and standard M5 threaded fluidic connectors for pressure-tight connections to tubings or capillaries. The overall size of the pump is 28 mm × 30 mm × 24 mm. It has a total internal volume of 4.12 mL and an active pumping volume of 2.58 mL. The complete design is illustrated in Figure 2 as half-cut side and top view.

### 2.3. Electromagnetic Actuation

**Figure 3:**
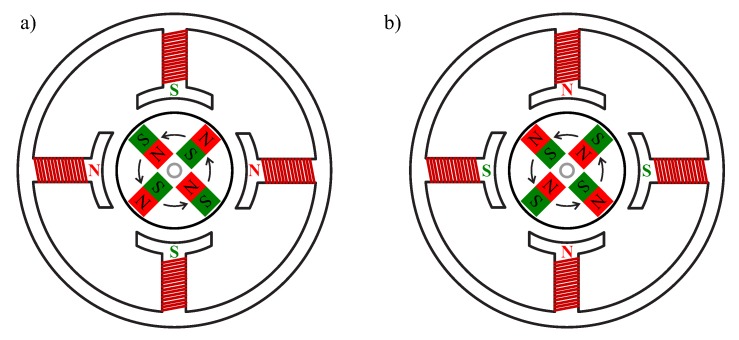
Principle of electromagnetic actuation of permanent magnets embedded in rotor (middle) through switching of the current direction through stator coils between state (**a**) and (**b**).

For actuation, the pump requires no mechanical or electrical connections into the rotor chamber. Instead, the magnets embedded in the rotor are electromagnetically pushed and pulled by external stator coils. The design is based on a brushless DC motor, specifically as an inrunner with the rotor located inside the stator. Complementary to the four embedded magnets we utilize four stator coils, which are connected in series and therefore electrically act as one large inductance $L_{coils}$. The direction of the coil winding is alternating from coil to adjacent coil, similar to the polarity of the embedded permanent magnets, resulting in alternating directions of the magnetic fields. Figure 3 illustrates the complete arrangement. By using an additional permanent magnet outside the pump, the rotor position is initialized to an approximately $45^{\circ }$ offset to the stator, for example, as in Figure 3a. Running a current through the stator coils leads to opposing magnetic poles attracting each other and vice versa. This results in a rotation of the rotor.

**Figure 4:**
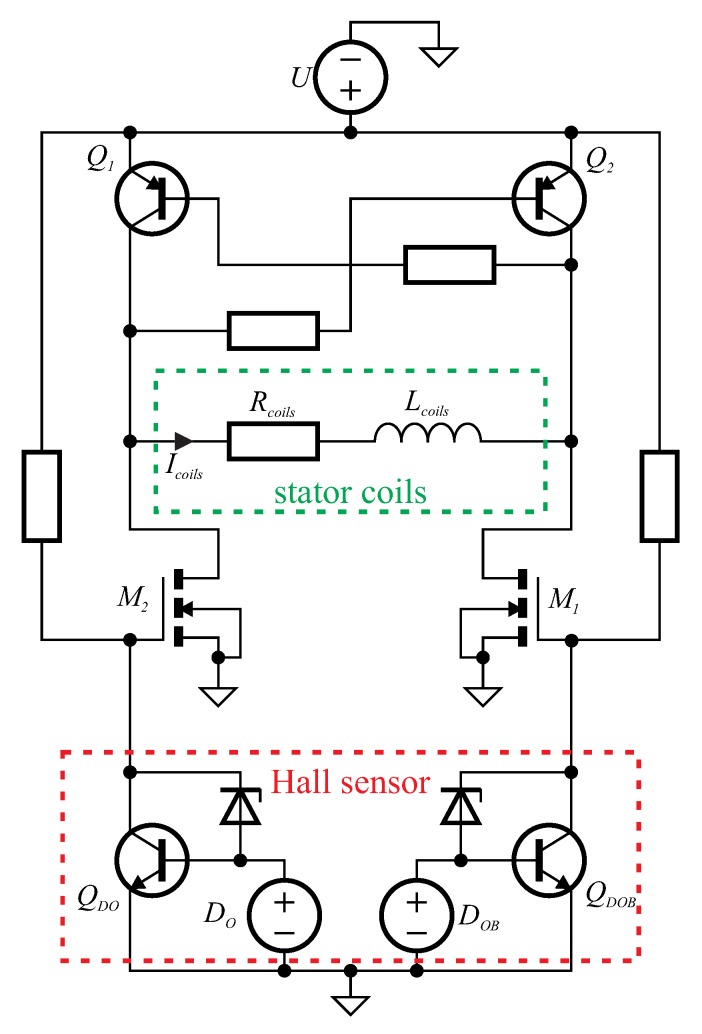
Schematic of coil current control circuit, with Hall sensor IC (given as schematic principle) switching two metal-oxide-semiconductor field-effect transistors (MOSFETs) $M_{1}$ and $M_{2}$ enabling current $I_{coils}$ to flow in one or the other direction from driving PNP transistors $Q_{2}$ and $Q_{1}$ through the stator coils.

After stator coils and corresponding rotor magnets reach their minimal distance, the current direction through the coils is switched so that the now same magnetic poles repulse each other and the magnets are attracted to the next coil (Figure 3b). To achieve a sustained and stable rotation, the current switching has to be timed accurately. To that end, we place a Hall sensor flip-flop IC (*AH277A*, BCD Semiconductor Manufacturing Ltd., Shanghai, China) with complementary outputs along the inner circumference of the stator between the poles of two coils to measure the position of the rotating magnets. A change in the direction of the strong magnetic field generated by the permanent magnets leads the IC to switch its outputs. The complete electrical driving circuit is given in Figure 4.

We connect the Hall sensor IC (dashed red schematic representing the working principle in Figure 4) to a custom amplification circuit comprising two arms. The Hall sensor switches two high power n-type metal-oxide-semiconductor field-effect transistors (N-MOSFETs), $M_{1}$ and $M_{2}$, which separate the IC from the high driving current through the stator coils (dashed green). The coil current $I_{coils}$ is driven by two corresponding bipolar junction PNP transistors, $Q_{2}$ and $Q_{1}$ respectively, in one or the opposing direction depending on which MOSFET is switched to its conducting state. Precise positioning of the Hall sensor IC is critical to achieve the correct timing and therefore a sustained rotation.

### 2.4. Fabrication and Assembly

**Figure 5:**
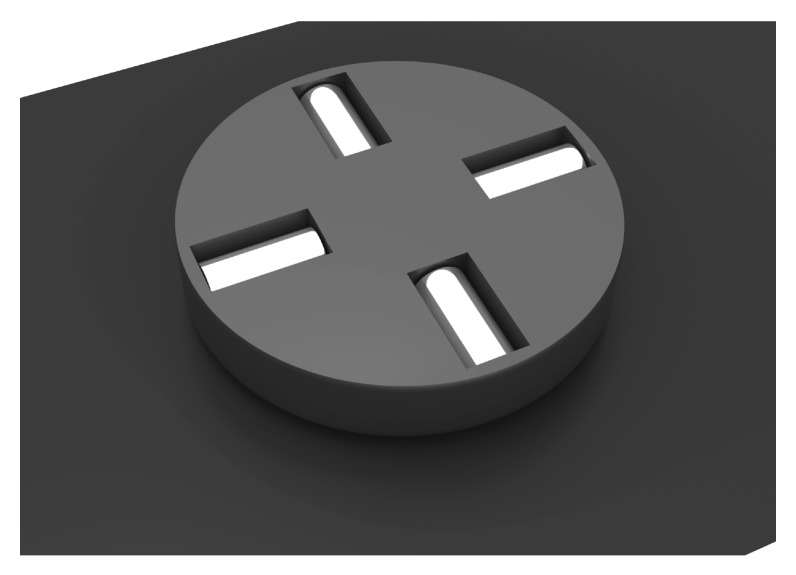
Principle of permanent magnet insertion by pausing printing process at last layer of magnet cavities and before printing closing layer, with magnets alternating in magnetization direction.

The three pump components (rotor, housing and lid) are fabricated using a microstereolithography high-resolution digital light processing (DLP) printer (*MAX X27 UV*, Asiga, Sydney, Australia) utilizing a 385 nm ultraviolet (UV) light-emitting diode (LED) to selectively solidify a high-precision photopolymer resin (*FusionGRAY*, Asiga, Sydney, Australia) or a transparent microfluidic resin (*MF BV007*, 3DXS GmbH, Erfurt, Germany) with a spatial resolution of 27 µm and a slicing thickness of 25 µm. A critical step is the printing of the rotor. We stop the print after the last layer creating the magnet cavities, remove the platform from the printer and manually embed cylindrical permanent NdFeB magnets with a diameter of 2 mm and a length of 4 mm in alternating orientations using a custom printed insertion tool. Afterwards, the platform is reattached, carefully aligned to the same position as prior to pausing and printing resumes with the next layer covering the magnets. In our final design and fabrication, we observe no faults or layer adhesion problems due to this print-pause-print integration process. We also do not remove the liquid resin from the magnet cavities during embedding, so that the magnets are firmly integrated in the material after curing. The embedding step is illustrated in Figure 5.

**Figure 6:**
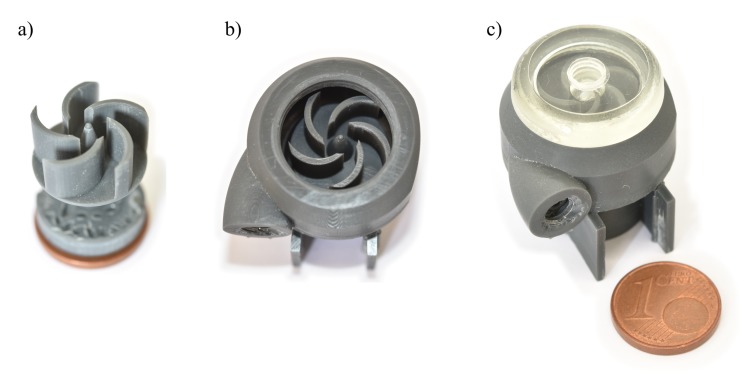
Photographs of (**a**) printed rotor with embedded magnets, (**b**) top view into pump chamber with inserted rotor and (**c**) complete pump with closed, transparent inlet lid and output connector on front. The overall width, depth and height of the pump is 28 mm × 30 mm × 24 mm, respectively.

For post-processing, first all components are thoroughly rinsed in isopropanol. Final cleaning is done using a standard ultrasonic bath for at least two minutes. Support structures are then removed manually with a scalpel and tweezers and everything is dried using a nitrogen flow. Finally, the parts are cured in a standard flood UV chamber for 5–10 min. Finished parts are immediately usable. Photographs of the fabricated parts are shown in Figure 6.

The stator ring is printed via filament extrusion (*Sigma*, BCN3D Technologies, Barcelona, Spain) using a ferromagnetic material with a relative magnetic permeability of 5–8. Ferromagnetic M3 screws make up the cores of the stator coils to increase magnetic field strength and assist with coil winding. The diameter of the stator is 58 mm with a height of 7.3 mm. Approximately 480 turns are alternatively wound around each of the four cores using an enameled copper wire with a diameter of 0.15 mm. This results in a typical outer coil diameter of 9.5 mm, a length of 5–6 mm and a total resistance of all four coils of $R_{coils}=10\;\Omega$. The stator is fixed on a custom circuit board together with the Hall sensor IC and driving circuit, while the pump is placed inside the stator but can be freely removed or exchanged. The complete board with stator and pump covers a size of approximately 10 cm × 8 cm.

### 2.5. Measurement Setup

**Figure 7:**
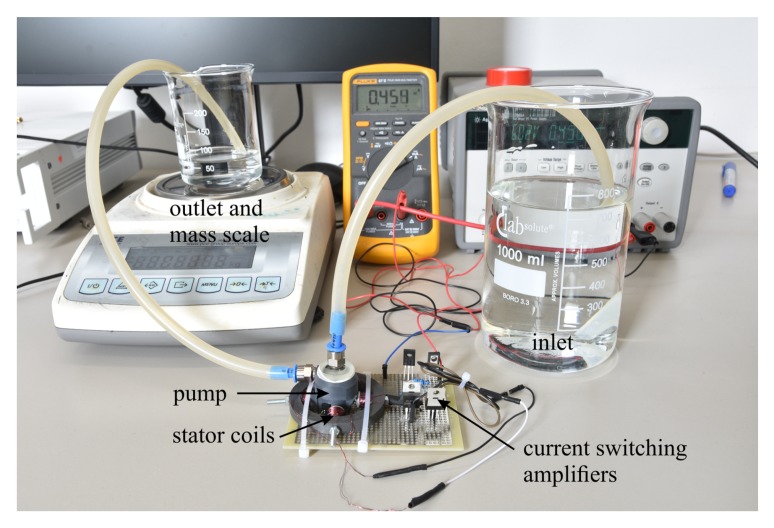
Photograph of experimental setup with printed, centrifugal pump placed inside stator coils on current switching circuit board (bottom center), board driven by laboratory power supply with applied current measured with a multimeter (back), inlet reservoir and outlet reservoir on similar liquid level to prevent hydrostatic flow, output flow measured with mass scale (left).

For experimental characterization of the pump, we connect it to inlet and outlet reservoirs of deionized (DI) water via differently sized fluidic tubings. Stator coils and circuit are supplied by a variable laboratory power supply with the applied input current measured by a multimeter. A photograph of the complete setup is shown in Figure 7.

Additionally, the rotation speed of the pump is calculated from the current switching frequency, with two periods or four switches per revolution. Similar to large-scale centrifugal pumps, the chamber and tubings need to be filled with liquid prior to operation. Imperfect filling can leave residual air bubbles stuck in the pump, which can decrease output performance. To avoid this critical issue we repeatedly push liquid through the pump until we observe the removal of air and complete filling through the transparent lid. For determination of the flow rate, we measure the added mass in the outlet reservoir with a precision scale (*PCE-BT 200*, PCE Deutschland GmbH, Meschede, Germany) within a specified time. The liquid levels of inlet and outlet container are adjusted to the same height prior to each measurement, in order to prevent any hydrostatic flow in either direction. To account for larger flow rates and subsequent larger changes in the liquid levels within the measurement time frame, the height of the outlet level is decreased by half the expected volume to compensate positive and negative influence of hydrostatic flow. The achievable output pressure has been determined by measuring the maximum height of pumped liquid relative to the input level.

The experimental setup depicted in Figure 7 allows observation of the electromagnetic actuation principle and characterization of maximum flow rate and output pressure generated by the 3D-printed centrifugal pump. Additionally, we connect a laminar micromixer to demonstrate using this pump in milli- and microfluidic applications.

## 3. Results and Discussion

### 3.1. Volumetric Flow Rate

**Figure 8:**
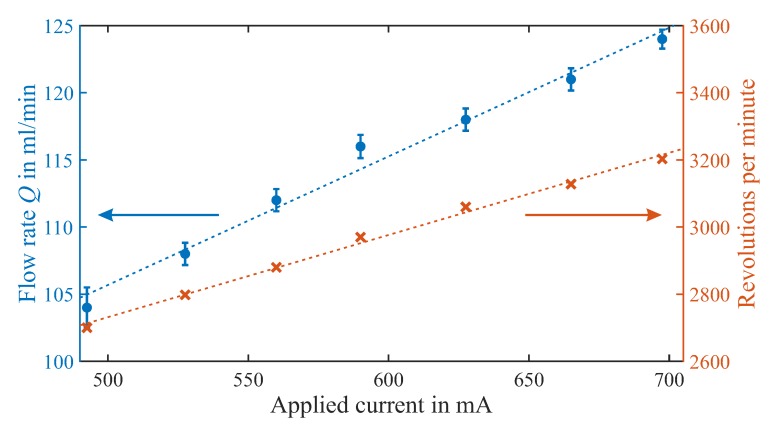
Measurement results for average flow rate *Q* (blue dots, left axis) and rotation speed (orange crosses, right axis) plotted over applied current, with dashed lines as linear fit.

The first characteristic of a pump is the achievable flow rate. To ensure repeatability and increase accuracy, four measurement runs are averaged and the standard deviation is included in the results. The added mass is measured after a pumping time of 1 min for each data point. In Figure 8 we plot flow rate *Q* and rotation speed over applied current for a driving voltage *U* ranging from 6–9 V. Here, the connected tubings are relatively large, with an inner diameter of 5 mm. This yields a relatively low fluidic resistance and the result is therefore indicative of the maximum achievable flow rate with this pump design. The measured flow rate (blue dots) increases linearly from 103 ± 1.7% mL/min to a maximum of 124 ± 0.7% mL/min at an input current of 700 mA. Considering the four repetitions, we can see a very low standard deviation of the average flow rates. The primary critical issue is air bubbles getting stuck in the pump, which can decrease the output performance. The rotation speed (orange crosses) also increases linearly with driving current from 2700–3200 rpm.

Decreasing the size of the connected tubings increases the fluidic resistance and therefore decreases the achievable flow rate. For an inner tubing diameter of 2 mm we measure an average flow rate of 25.3–34 mL/min for a similar driving voltage of 6–9 V, at slightly lower applied current and rotation speed of approximately 2500–3000 rpm. As expected for a centrifugal pump, the output flow rate varies with the system pressure.

### 3.2. Output Pressure

**Figure 9:**
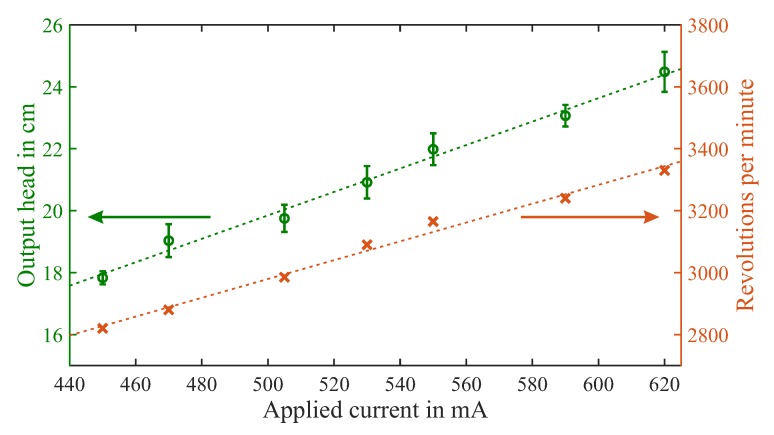
Measurement results for output pressure head (green circles, left axis) and rotation speed (orange crosses, right axis) plotted over applied current, with dashed lines as linear fit.

The achievable output pressure is independent of the connected fluidic resistance as it is a static state. In Figure 9 we plot the static output pressure head relative to the input level and rotation speed over applied current. The corresponding driving voltage again varies from 6–9 V. The average value and standard deviation of six individual measurement runs are given. We also investigated an influence of the placement of the pump above and below the input liquid level but found no differences. output pressure (green circles) also rises linearly with driving current, in line with the rotation speed (orange crosses). We measure a pressure head ranging from 17.8 ± 1.3% cm to 24.5 ± 2.9% cm. From the Bernoulli equation this corresponds to a hydrostatic pressure of 1750–2400 Pa, respectively.

### 3.3. Comparison with other 3D-Printed Pumps

Our 3D-printed centrifugal pump performs as expected, achieving very high flow rates at reasonable pressure levels. In Table 1 we compare the performance characteristics with selected examples from literature of micropumps and other miniature 3D-printed pumps. As reference and for size comparison and scaling, we list the total pump chamber volume. Examples for displacement pumps include a miniaturized pneumatic check-valve pump [13] and a rotary micro-gear pump [14], along with the 3D-printed peristaltic and membrane pumps [7,8,15]. These types typically offer very high maximum pressures at mediocre flow rates. Dynamic pumps such as a MEMS electrohydrodynamic pump [16], the 3D-printed Tesla pump [9], as well as the centrifugal pump from this work yield very high flow rates at lower output pressures. Compared to the similar sized rotary Tesla pump, our 3D-printed device features ten times higher maximum flow rate and output pressure. If we scale the flow rate by the pumping volume, our device performs in a similar range as the considerably smaller printed pneumatic pumps.

### 3.4. Pumping in a Microfluidic Channel and Outlook

**Figure 10:**
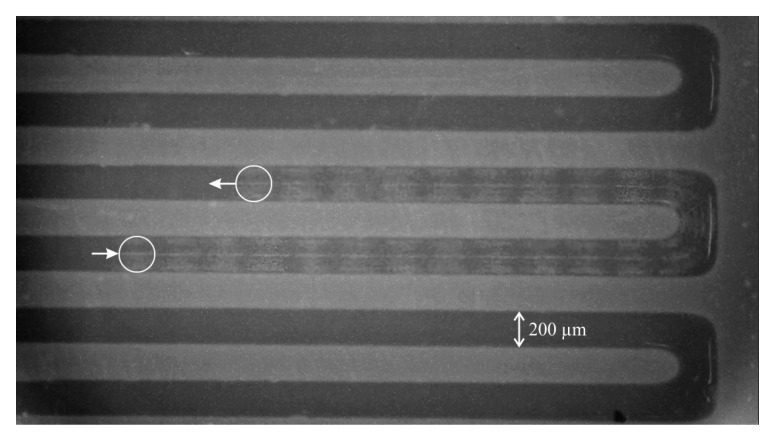
Dark-field microscope image of a laminar micromixer with a meander channel of 200 µm width, with 25 video frames (1 s) superimposed to illustrate movement of an oil droplet in the water flow driven by the centrifugal pump.

To demonstrate the suitability of this centrifugal pump for milli- and microfluidic applications, we connect it to one inlet of a laminar micromixer. The main meander channel of the mixer is 200 µm wide and 500 µm deep. We connect a syringe containing oil to the second inlet to generate oil droplets in the water stream from the pump to visualize the flow. A real-time microscope video of the flow of an oil droplet in a water stream driven by the centrifugal pump is available as Supplementary Video S1. Figure 10 is a dark-field microscope image with 25 individual frames (1 s) from that video superimposed to illustrate the movement of an oil droplet in the meandering channel. The droplet position in the first and last frame are marked with a white circle.

The overall volume of our pump can still be reduced in different ways. The current design and height of the rotor leaves a considerable amount of dead volume in the pumping chamber, which can also be problematic for air bubbles getting stuck inside and reducing performance. Closing the distance between impeller and embedded magnets is possible. The size of outlet and inlet can also be reduced to smaller connectors. However, the main limiting factor for further miniaturization is the available size of permanent magnets and manually embedding them. A strong magnetic field is required for the rotor and NdFeB magnets typically do not come in dimensions smaller than 1 mm. They can also not be placed arbitrarily close together due to the attractive and repulsive forces intrinsic to the magnet alignment. Fabrication of smaller designs is no issue with microstereolithography printing, as much finer details below 100 µm can be realized. In terms of electromagnetic actuation, stator coils and driving circuit can be improved to handle higher currents and a larger operation range. This would mainly require a better Hall sensor IC to more accurately and reliably detect the magnet rotation and switch the current accordingly, as well as solutions for improved heat dissipation of stator coils and driving transistors. Finally, combining the stator with a suitable, printed housing to optimally align the pump and to include the electronics is a matter of ongoing development.

## 4. Conclusions

We have presented a miniature 3D-printed centrifugal pump for very high flow rate applications of millifluidic and microfluidic devices. Permanent magnets integrated during the printing process allow for non-contact electromagnetic actuation of a free-moving rotor inside the pump housing. Using microstereolithography printing we have reduced the overall size of this dynamic pump to 28 mm × 30 mm × 24 mm with an active pumping volume of 2.58 mL. Customized stator coils and switching circuitry based on a Hall effect sensor complete the portable device package.

Our experimental characterization demonstrates a very high flow rate up to 124mL/min, which is highly dependent on the connected fluidic resistance. For a dynamic pumping principle, we measure a reasonably high hydrostatic pressure of up to 2400 Pa. Further miniaturization of the pump design is possible but limited by the size of high-strength permanent magnets necessary for non-contact actuation. We believe our work illustrates the feasibility of using additive manufacturing to realize miniature versions of macroscale pumping concepts not previously investigated for applications with microfluidic systems.

## Figures and Tables

**Table 1:** Comparison of selected miniaturized and 3D-printed pump types with this work, estimating pump chamber volume and giving maximum flow rate *Q* and maximum output pressure.

Pump Type	Volume/µL	Max. *Q*/mL·min^−1^	Max. Pressure/Pa	Ref.
Pneumatic check-valve	≈15.4	13	5900	[13]
Rotary micro-gear	≈0.14	0.055	12,500	[14]
Electrohydrodynamic	≈3.42	14	2480	[16]
Magnetohydrodynamic	0.19	0.0015	180.5	[17]
Peristaltic (3D-printed)	74.8	0.68	–	[7]
Membrane (3D-printed)	0.495	0.02	24,130	[8]
Tesla (3D-printed)	≈3380	12	253	[9]
Peristaltic (3D-printed)	120	0.71	≈56	[15]
Vibrating blades (3D-printed)	≈9000	107.8	–	[18]
**Centrifugal (3D-printed)**	≈4120	124	2400	**this work**

## Supplementary Materials

The following are available online at https://www.mdpi.com/2072-666X/10/10/631/s1, Video S1: Real-time dark-field microscope video of oil bubble moved by water stream from centrifugal pump through 200 µm wide meander channel of a laminar micromixer.
